# Dynamics of Aboveground Natural Enemies of Grasshoppers, and Biodiversity after Application of *Paranosema locustae* in Rangeland

**DOI:** 10.3390/insects10080224

**Published:** 2019-07-26

**Authors:** Wang-Peng Shi, Xiao-Yu Wang, Yue Yin, Yu-Xing Zhang, Um-e-Hani Rizvi, Shu-Qian Tan, Chuan Cao, Hong-Yan Yu, Rong Ji

**Affiliations:** 1 College of Life Science, International Cooperative Research Centre for Cross-border Pest Management in Central Asia, Xinjiang Normal University, Urumqi 830054, China; 2 Department of Entomology, China Agricultural University, Beijing 100193, China; 3 Qinghai Rangeland Work Station, Xining 810008, China

**Keywords:** *Paranosema locustae*, biodiversity, grasshoppers, biological control

## Abstract

Substantial harm to ecosystems from the use of chemical pesticides has led to an increasing interest in the use of biopesticides to control grasshoppers in rangelands, including China. One such potential biopesticide for control of grasshoppers is the fungus *Paranosema locustae*. In this study, the dynamics of aboveground natural enemies of grasshoppers and arthropod diversity 0–9 years after application of *P. locustae* were investigated in rangeland in Qinghai Plateau, China. We found that the number of species and of individuals of aboveground natural enemies increased by 17–250% and 40–126%, respectively, after spraying *P. locustae*, and that the main natural enemies showed three peaks after treatment. The conventional indices of species diversity (H’) and evenness (J’) increased by 11–267% and 13–171%, respectively, after treatment with *P. locustae*. The results showed the positive effects of *P. locustae* on aboveground natural enemies and biodiversity in an arthropod community in Chinese rangeland. *Paranosema locustae* is thought to be a safe biological control agent for grasshopper management in Northwestern China.

## 1. Introduction

More than 40 species of pest insects affect some or all of the 400 million hectares of grasslands in China. Of these species, grasshoppers are the most important, causing the largest losses in pastures and crops [1]. In an average year, more than 15–20 million hectares of grassland in northern China experience grasshopper outbreaks, and such outbreaks are common on the Qinghai–Tibet Plateau. These outbreaks have led to the need for continued research on biological control, because of the potential damage to the environment and rangeland ecosystems from the widespread use of chemical pesticides. The need for a biological alternative is especially important for the Qinghai–Tibet Plateau in China.

Lots of entomopathogens have been evaluated for grasshopper control, including *Paranosema locustae* Canning (Microsporidia: Nosematidae), *Metarhizium* spp [2], *Malamoeba locustae* (King et Taylor) [3], and *Beauveria bassiana* (Balsamo) *Vuillemin*. Meanwhile, *Metarhizium acridum* (Driver & Milner) J.F. Bisch., Rehner & Humber (Green Muscle^®^, Green Guard^®^) is effective in controlling the desert locust and Australian plague locust, *Chortoicetes terminifera* Walker [4]. More than 120 species of grasshoppers are susceptible to *P. locustae* [5]. The high spore production of *P. locustae*-infected hosts, the vertical transmission of the disease through its host, and its wide host range within acridid grasshoppers gives it the potential for wide application for controlling grasshoppers in grasslands.

Over the past twenty years, more than 1 million hectares have been treated with *P. locustae* in China, demonstrating the efficacy of this biological agent for managing grasshoppers [6]. *P. locustae* remains in grasshopper populations even in high ultraviolet rays’ area Qinghai Plateau for many years, and the density of primary grasshopper species was kept below EIL in these treated areas [7]. Predatory natural enemies are important vectors for this microsporidian disease [8]. A great deal of attention has been paid to the effects of the pathogen on non-target arthropods, especially on the main natural enemies of grasshoppers and insect biodiversity, in general, in rangeland. The current study examines the effects of *P. locustae* on the principal groups of aboveground natural enemies of grasshoppers and arthropod biodiversity in a rangeland arthropod community over a nine-year period following application in the Qinghai–Tibet Plateau.

## 2. Materials and Methods

### 2.1. Entomopathogen Formulations

*P. locustae* from North America was propagated at the China Agricultural University, Beijing, China, by inoculating laboratory-reared third instar *Locusta migratoria* with spores via oral inoculation in water at 1.0 × 10^5^ spores/mL. After rearing inoculated grasshoppers for 30–40 days, spores were collected from cadavers, with an average yield of 2.0 × 10^10^ spores per locust. The spores were formulated in water (1.0 × 10^10^ spores/mL water) and stored in a freezer [8]. When most grasshoppers in the field were in the third instar, formulated spores were applied as an ultra-low-volume spray using a Taishan 1806 mobile fogging machine (Taian Pesticide Machine Ltd, Taian, China). The formulated dose level was 7.5 × 10^9^ *P. locustae* spores in 12 L water/hm^2^.

### 2.2. Experimental Plots

The experimental sites covered an area of 15,000 hectares in Tianjun County (Altitude, 3417 m; 99°02′E; 37°18′N), Qinghai–Tibet Rangeland. There were three plots for both treated and control; every plot was 1500 hectares of natural pasture; and there were discrete areas separated by untreated terrain.

### 2.3. Sampling

For nine years after treatment with *P. locustae,* grasshoppers and aboveground main natural enemies [9], such as formicid ants, the carabid beetle *Aristochroa venusta* Tschitschérine, the scelionid wasp *Scelio nikolski*, the meloid *Epicauta gornami* Marseul, the sarcophagid fly *Sarcophaga filipievi* (Rodhain), the bombyliid fly *Systoechus* sp., spiders, and the agamid lizard *Phrynocephalus vlangalii* Strauch were collected from the treated and control area at the end of August every year; the initial time of 0 is the finding for the year of *P. locustae* application. The different stage of natural enemies and arthropods above described were collected with an insect suction collector (UNIVAC, Burkard Agronomics, England) at all treated and untreated plots [10], and insect densities were estimated within square aluminium frames (length × width × height: 1 × 1 × 0.5 m), making 50 such collections to estimate insect density within each plot.

All insects were collected from the areas defined by the aluminium frames with suction provided by the D-vacs, and all collected material was placed individually by sample into plastic bags before being stored in a freezer for later identification of insect contents. For common species, including *Oedaleus decorus asiaticus* Bey-Bienko, *Dasyhippus barbipes* (F.-W.), *Bryodema luctuosum luctuosum* Stoll, *Angaracris barabensis* Pallas, and *Myrmeleotettix palpalis* Zubovski besides the natural enemies above described, the abundance (number of individuals) was noted and all specimens collected were identified.

### 2.4. Statistical Analysis

The conventional indices of species diversity (Shannon diversity, H’) and evenness (J’ = H’/*ln* (S’) [11] were used to compare the species richness and species diversity of arthropod communities of the Qinghai–Tibet Plateau region in the study area. The indices were calculated using data from a series of sample collections made in the study area. The variance of the Shannon index and evenness was calculated as per [12,13].

One-way ANOVAs were used to compare the density and rate of infection in grasshoppers between treated and untreated areas with least-significant difference (LSD) analysis used for multiple comparisons to measure the difference between different treatments. SPSS 17.0 was used for the calculations and analysis.

## 3. Results

### 3.1. Population Fluctuations of Grasshopper’s Natural Enemies

**Figure 1:**
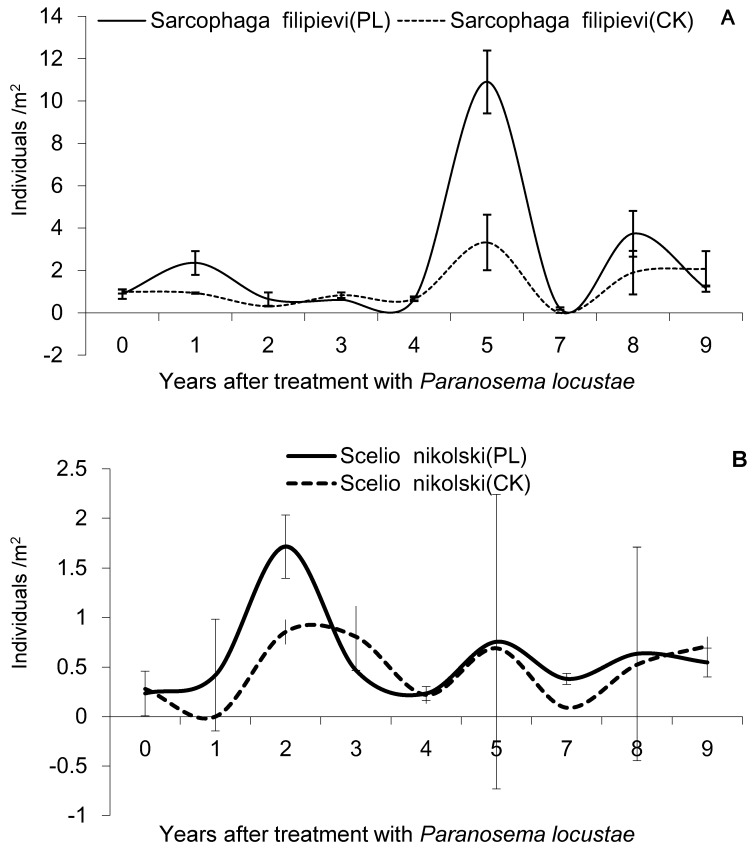

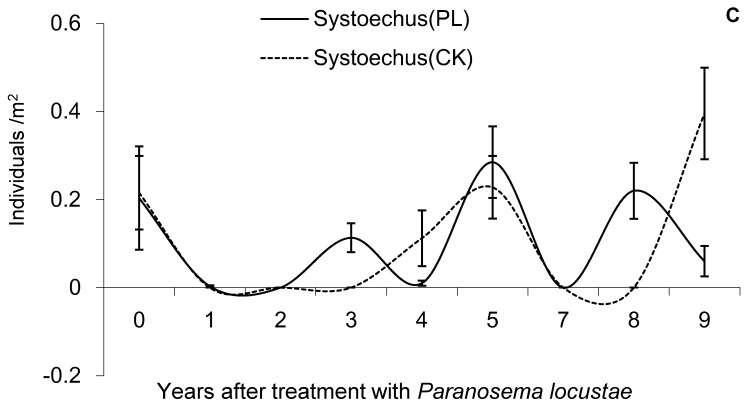
Densities of grasshopper parasitoids in study area, 0–9 years after treatment with *Paranosema locustae*.

The sarcophagid fly *S. filipievi* is one of the main parasitoid of grasshoppers, and three population peaks were observed for this species, in the first (2.35 ± 0.56 individuals/m^2^), fifth (10.90 ± 1.48/m^2^), and eighth (3.73 ± 1.07/m^2^) year after treatment with *P. locustae*, and the sum of all counts of *S. filipievi* showed significant difference for treatment versus control (*F* = 17.373, *df* = 8, 18, *p* < 0.05), but *S. nikolski* (*F* = 2.21, *df* = 8, 18, *p* > 0.05) and *Systoechus* (*F* = 1.454, *df* = 8, 18, *p* > 0.05) showed any insignificant differences between treatment and control, respectively (Figure 1).

Analysis (one-way ANOVA) of differences of the sum of all counts of parasitoids of grasshoppers between the control (CK) and treated area (PL)—A: *S. filipievi* (*F* = 17.373, *df* = 8, 18, *p* < 0.05); B: *S. nikolski*, (*F* = 2.21, *df* = 8, 18, *p* > 0.05); C: *Systoechus*, (*F* = 1.454, *df* = 8, 18, *p* > 0.05).

**Figure 2:**
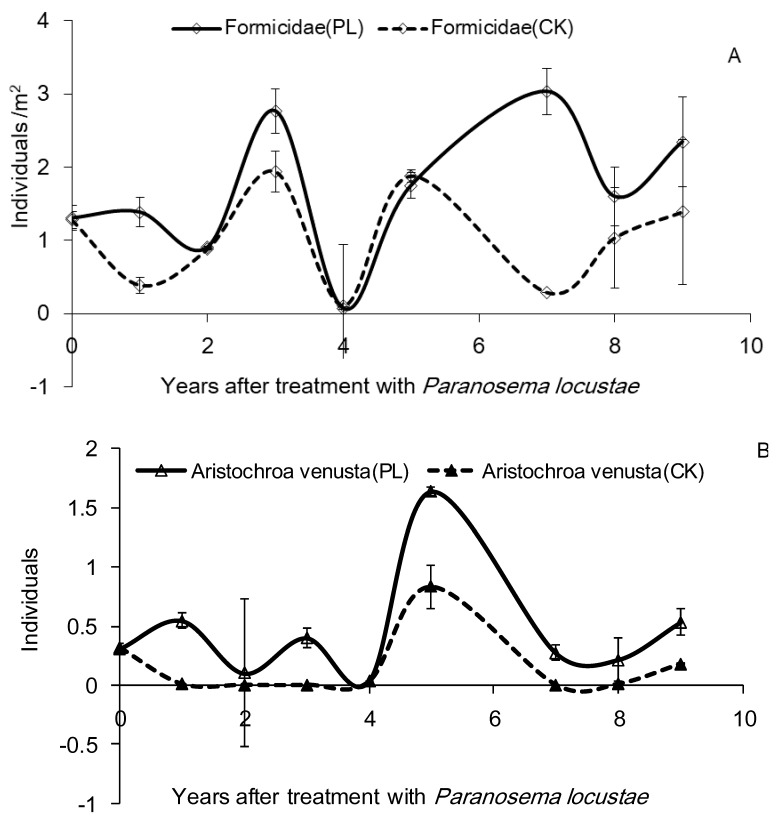

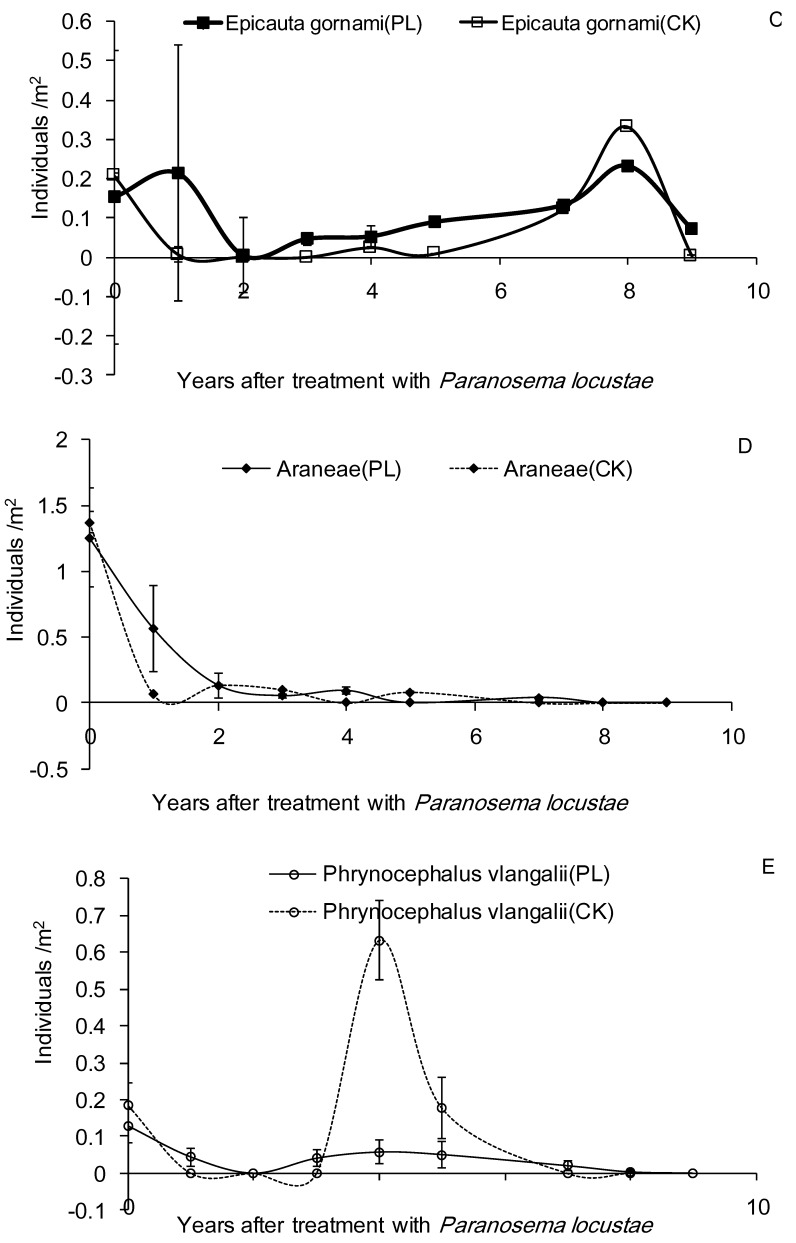
Densities of grasshopper predators 0–9 years after treatment with *P. locustae*.

A predator, the carabid beetle *A. venusta,* also showed three peaks, in the first (0.55 ± 0.16/m^2^), fifth (1.64 ± 1.03/m^2^), and ninth (0.53 ± 0.10/m^2^) year after treatment. Ants (Formicidae) also showed three peaks, in the third (2.77 ± 0.31/m^2^), seventh (3.03 ± 1.12/m^2^), and ninth (2.35 ± 0.61/m^2^) year after treatment, but the sum of all counts of Formicidae (*F* = 1.321, *df* = 8, 18, *p* > 0.05), *A. venusta* (*F* = 0.908, *df* = 8, 18, *p* > 0.05), *E. gornami* (*F* = 1.19, *df* = 8, 18, *p* > 0.05), Araneae (*F* = 2.158, *df* = 8, 18, *p* > 0.05), and *P. vlangalii* (*F* = 0.991, *df* = 8, 18, *p* > 0.05) showed no significant difference for treatment versus control, respectively (Figure 2).

Analysis (one-way ANOVA) of differences of the sum of all counts of predators of grasshoppers between the control (CK) and treated area (PL)—A: Formicidae (*F* = 1.321, *df* = 8, 18, *p* > 0.05); B: *Aristochroa venusta* (*F* = 0.908, *df* = 8, 18, *p* > 0.05); C: *Epicauta gornami* (*F* = 1.19, *df* = 8, 18, *p* > 0.05); D: Araneae (*F* = 2.158, *df* = 8, 18, *p* > 0.05); E: *Phrynocephalus vlangalii* (*F* = 0.991, *df* = 8, 18, *p* > 0.05).

### 3.2. Diversity of Arthropods after Application of P. locustae

**Figure 3:**
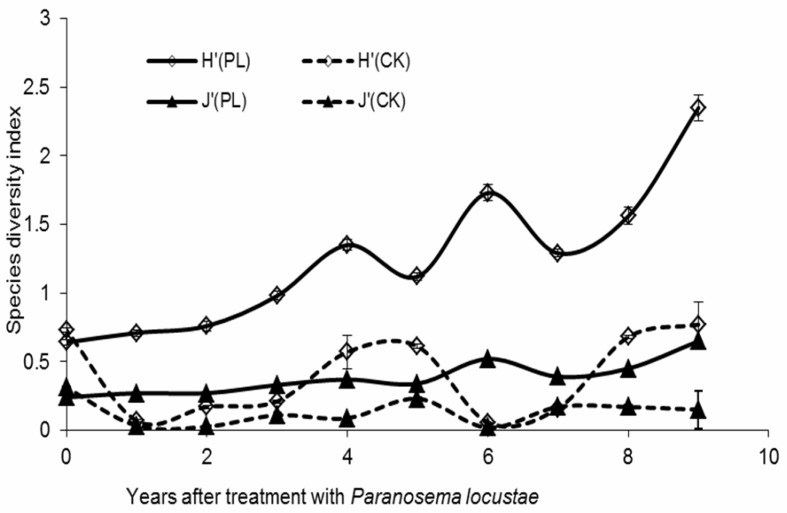
Arthropod richness and evenness indices, 0–9 years after treatment in plots treated with *P. locustae*.

Nine years after treatment with *P. locustae*, the total number of species and individuals of all the named species collected (see above described) increased by 250% and 126%, respectively, in areas treated with *P. locustae*. At the same time, the conventional indices of species diversity (H’) and evenness (J’) increased by 267% and 171%, respectively. Analysis of overall data showed that there were significant differences between in the control (CK) and treated area (PL) for both the Shannon index for diversity (H’) and evenness (J’) (*F* = 14.647, *df* = 9, 50, *p* < 0.05) and evenness (J’) (*F* = 7.354, *df* = 9, 50, *p* < 0.05) (Figure 3).

Analysis (one-way ANOVA) of overall differences of diversity indices of arthropod between the control (CK) and treated area (PL): the Shannon index for diversity (H’) (*F* = 14.647, *df* = 9, 50, *p* < 0.05) and evenness (J’) (*F* = 7.354, *df* = 9, 50, *p* < 0.05).

### 3.3. Fluctuations of Grasshoppers among Years 0–9 Post-Treatment

**Figure 4:**
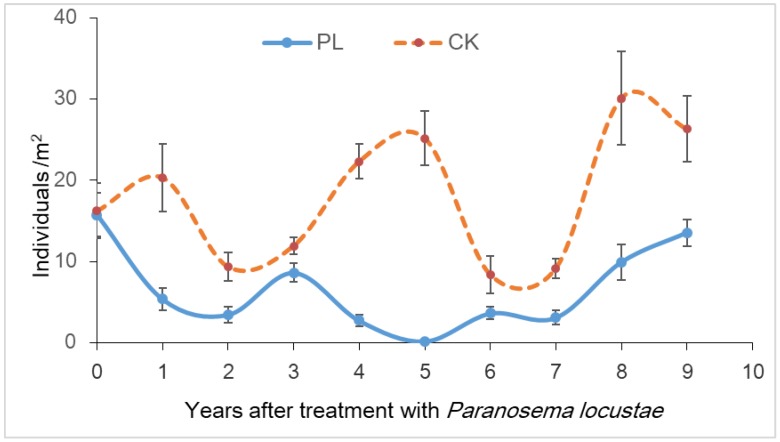
Mean densities ± SE (vertical bars) of grasshoppers in the study area, 0–9 years after treatment with *P. locustae.*

At the time of treatment, grasshoppers were 15.76 ± 2.67 individuals/m^2^, but it declined by 98.9% by the fifth year after treatment with *P. locustae*. Grasshoppers increased after the sixth year and peaked to 13.56 ± 1.64 in year nine (Figure 4). Analysis (one-way ANOVA) of differences of the sum of all counts of grasshoppers between the control (CK) and treated area (PL) (F = 11.221, *df* = 9, 20, *p* < 0.05) was done.

Analysis (one-way ANOVA) of differences of the sum of all counts of grasshoppers between the control (CK) and treated area (PL) (*F* = 11.221, *df* = 9, 20, *p* < 0.05) was performed.

## 4. Discussion

*P. locustae* has been found to be a useful tool of grasshopper management in the rangeland [1,6]. Although *P. locustae* often causes only low-to-moderate levels of direct mortality post-treatment [14], the cumulative usefulness of *P. locustae* can be far greater [6,15]. In the present study, we found increases in both the number of species and the number of individuals of natural enemies of grasshoppers in the study area over the nine-year period after the application of *P. locustae*, demonstrating an indirect benefit for controlling grasshoppers with this bio-agent.

In addition to the level of grasshopper mortality obtained using products like *P. locustae*, grasshopper management programs should consider the sustainability of their practices and long-term effects on the health of the rangeland ecosystem [1]. A similar impact of *P. locustae* on a rangeland system was observed in Xinjiang and Inner Mongolia provinces and in Argentina [15,16], but the causes of the long-term effects post-treatment with *P. locustae* require further research.

In plots treated with chemical pesticides in Tianjun, the carabid beetle *A. venusta* and the lizard *P. vlangalii* numbers declined by about 70% and 35%, respectively, one year after treatment, compared to areas treated with *P. locustae*, and the chemically treated plots had to be re-treated with pesticides after 3–4 years [1]. An important concern for the sustainable use of biological pesticides over wide areas is their effect on non-target native arthropods, especially natural enemies of the targeted pest. Arthropods are the most diverse group of organisms in ecosystems and include many species with biodiversity or pest control value. Here, we used diversity assessments of the natural enemy assemblages in *P. locustae*-treated areas to evaluate the impact of *P. locustae* on the main natural enemies and diversity in the study region. Although ants are highly diversified and exhibit several alimentary behaviors, most species of ants are to a greater or lesser degree predators or scavengers, and these ants we investigated like to feed on dead, dying, sick or molting grasshoppers in the grassland; so they were considered as a kind of natural enemy of grasshoppers in the experiment. We found that the common grasshopper natural enemies (Ants, *A. venusta*, *S. nikolski*, and *S. filipievi*) in the area were abundant, and that the conventional indices of species diversity (H’) and evenness (J’) showed significant increases, suggesting that *P. locustae* can be considered a safe biological agent to non-target arthropods, and its use advocated in grasshopper management. Another one, the infected grasshopper was easy to be predated by its predators because of slow behavior, which is beneficial for natural enemies of grasshoppers. The predatory natural enemies of grasshoppers are important vectors for *P. locustae*, so the increases of natural enemies of grasshoppers improve the spread of the pathogen in the field. The density of Araneae has suffered a fall over the nine-year period, and we think that the rainfall and humidity declines have been detrimental to spider in recent years. The impact of *Paranosema* to native entomopathogeny of grasshopper will be researched further in the future.

The sustained control effect of *P. locustae* on grasshoppers was demonstrated here; the increases of natural enemies of grasshoppers perhaps are a potential factor; *P. locustae* has been found persisting in grasshopper populations many years after application in rangeland [16]; and the persistence of the pathogen also is helpful for long-term control of grasshopper, because the common grasshoppers are susceptible to *P. locustae* collected in the grassland [7].

## 5. Conclusion

The number of species and individuals of aboveground main natural enemies of grasshoppers and biodiversity of arthropods increased in regions treated with *P. locustae.* Preventive control of grasshoppers with biological pesticides *P. locustae* has been beneficial for keeping biodiversity of rangeland system.
